# Artificial Intelligence-Assisted Breeding for Plant Disease Resistance

**DOI:** 10.3390/ijms26115324

**Published:** 2025-06-01

**Authors:** Juan Ma, Zeqiang Cheng, Yanyong Cao

**Affiliations:** Institute of Cereal Crops, Henan Academy of Agricultural Sciences, Zhengzhou 450002, China; zeqiangcheng@163.com

**Keywords:** artificial intelligence, deep learning, large language model, plant disease, phenomics, phenotype

## Abstract

Harnessing state-of-the-art technologies to improve disease resistance is a critical objective in modern plant breeding. Artificial intelligence (AI), particularly deep learning and big model (large language model and large multi-modal model), has emerged as a transformative tool to enhance disease detection and omics prediction in plant science. This paper provides a comprehensive review of AI-driven advancements in plant disease detection, highlighting convolutional neural networks and their linked methods and technologies through bibliometric analysis from recent research. We further discuss the groundbreaking potential of large language models and multi-modal models in interpreting complex disease patterns via heterogeneous data. Additionally, we summarize how AI accelerates genomic and phenomic selection by enabling high-throughput analysis of resistance-associated traits, and explore AI’s role in harmonizing multi-omics data to predict plant disease-resistant phenotypes. Finally, we propose some challenges and future directions in terms of data, model, and privacy facets. We also provide our perspectives on integrating federated learning with a large language model for plant disease detection and resistance prediction. This review provides a comprehensive guide for integrating AI into plant breeding programs, facilitating the translation of computational advances into disease-resistant crop breeding.

## 1. Introduction

Plant diseases are major factors responsible for crop yield losses worldwide, which threaten global food security. Early identification, prediction and selection of disease-resistant traits are crucial to prevent yield loss. Traditional disease detection methods mainly rely on artificial observation and chemical analysis techniques [1]. These methods are laborious, destructive, and easily affected by human factors. The construction of rapid, accurate, efficient, and large-scale detection, and selection technologies is crucial in plant breeding for disease resistance, as it enables timely prevention and control of disease outbreaks, as well as rapid screening of disease-resistant germplasms.

**Figure 1:**
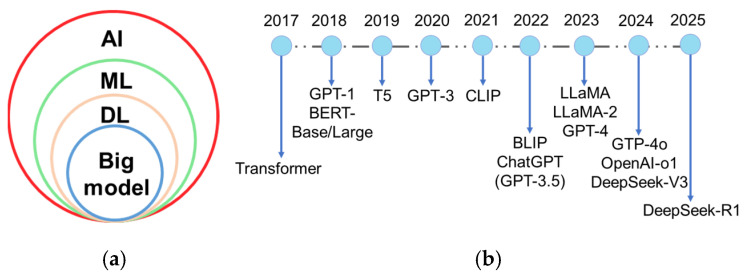
(**a**) The relationships among different artificial intelligence (AI) methods. ML: machine learning; DL: deep learning; (**b**) A brief history of big model. The figure illustrates the evolution of large language model and large multi-modal model from 2018 to 2025, beginning with the foundational breakthrough of the transformer architecture in 2017. It highlights major milestones like generative pre-trained transformer (GPT-1), bidirectional encoder representations from transformers (BERT-Base/Large, 2018), contrastive language-image pretraining (CLIP, 2021), bootstrapping language-image pre-training (BLIP, 2022), large language model meta AI (LLaMA/LLaMA-2, 2023) and GPT-4 (2023), and DeepSeek-V3/R1 (2024, 2025). B5: text-to-text transfer transformer.

Plant breeding has evolved from Breeding 1.0 to Breeding 4.0. Wallace et al. (2018) characterized Breeding 1.0 as incidental farmer-led selection, Breeding 2.0 as the application of statistical methods and experimental design to enhance selection efficiency, and Breeding 3.0 as the integration of genetic and genomic data-driven approaches [2]. Breeding 4.0, catalyzed by advances in genetics and information technologies [2], is driven by artificial intelligence (AI), multi-omics big data, and genome editing technologies. AI is the simulation of human intelligence by machines, which involves learning, perception, reasoning, prediction, and self-correction [3]. AI algorithms encompass machine learning (ML), deep learning (DL), and cutting-edge big models (Figure 1a). ML technology especially captures non-linear relationships between genotypes and phenotypes [4], overcoming the limitations of traditional linear regression models. DL, a subfield of ML based on artificial neural networks, can automatically extract data features and is well-suited for handling unstructured data. In particular, convolutional neural network (CNN), a key DL algorithm, excels at processing complex, large-scale, and high-resolution images. The introduction of the transformer model by Google researchers and collaborators in Attention Is All You Need [5] pioneered the era of large-scale AI models. Big model or foundation model typically refers to DL models with a massive number of parameters and trained on large-scale datasets, which enables them to handle complex tasks and excels in multiple domains [6,7]. A large language model (LLM) is a subset of big model, specializing in natural language processing tasks. LLM represents one of the most active and successful research areas in AI today. Beyond LLM, the big model also includes a large vision model (LVM) and large multi-modal model (LMM), which extends capabilities to visual and cross-modal understanding. A brief history of these models-encompassing key LLM, LVM, and LMM advancements-is illustrated in Figure 1b. These AI methods and their hybrid models have been widely utilized to identify diseases, classify diagnoses, and assess severity from images and other inputs [8,9,10]. With the aid of AI, farmers can perform real-time field monitoring, and acquire intelligent early warning, and effective plant protection practices. Despite technological advancements, human-centered AI design [11] must be prioritized in smart agriculture. Farmers can bring experience and prior knowledge understanding to the AI pipeline and enhance the performance and reliability of AI systems [11].

Screening germplasms for disease resistance is an important goal in plant breeding, as it helps develop varieties with improved resilience to pathogens and reduce crop losses. The conventional approach of screening plants for disease-resistant phenotypes is labor-intensive and costly. Genomic selection (GS) allows for the selection of genotype candidates in early generation based on genomic estimated breeding values in the breeding process, significantly revolutionizing plant breeding in the past two decades. GS has the potential to enhance genetic gain and shorten the breeding cycle for complex agronomic traits such as disease resistance which are challenging to phenotype. Numerous studies have demonstrated the effectiveness of linear models in capturing genetic additive variation for disease resistance, such as Fusarium ear/stalk rot, Fusarium head blight, rust, wilt, and spot in crops including maize, wheat, potato, sugarcane, and strawberry [12,13,14,15,16]. In contrast, ML and DL methods are known to capture nonadditive effects and have been successfully applied in genomic prediction for diseases such as stripe rust, blast, Fusarium head blight, sheath blight, black-streaked dwarf virus, smut, and Pachymetra root rot in rice, wheat, maize, barley, and sugarcane [17,18,19,20,21,22,23]. Some of these studies have demonstrated that ML and DL methods perform equally well or better than traditional linear statistical methods for predicting disease-resistant traits [19,20,21,22].

Phenomic selection (PS) was first proposed by Rincent et al. (2018) as an alternative to marker-assisted selection, utilizing near-infrared spectroscopy (NIRS) data for traits prediction in wheat and poplar [24]. Except for ground-based high-throughput phenotyping (HTP) technology, phenomic data can be acquired by leveraging the technological advancements in unmanned aerial vehicle (UVA) and satellite remote sensing platforms. PS employs AI algorithms to analyze HTP features, like vegetation indices and texture features from drone and satellite images. High-throughput multispectral or hyperspectral UAVs have been widely used for phenomic prediction in agriculture and forestry. These technologies enable rapid, non-destructive monitoring of key traits such as yield, dry matter yield, plant height, growth rates, protein context, and disease resistance (e.g., rust) in crops including maize, wheat, soybean, rye, and potato [25,26,27,28,29,30,31,32,33]. These studies have demonstrated that by leveraging AI models, PS enables more accurate identification of superior genotypes, making it a promising tool for practical breeding.

Disease phenotypes are comprehensive outputs of interconnections of biological processes involving multi-omics response to pathogen stress in plant development. Except for genetic markers or phenomic information, endophenotypes such as transcriptome, metabolome, microbiome, etc., can be used or integrated to predict disease resistance using AI-based predictive algorithms. In particular, DL has emerged as one of the most effective approaches in multi-omics data analysis due to its capability to capture nonlinear and hierarchical features through multi-layered neural network architectures [34,35]. These aspects mentioned above represent key areas of AI applications in enhancing plant disease resistance. While distinct, these areas are interconnected and complementary, collectively contributing to improved plant health, yield, and resilience.

In this article, we present a system review of AI advancements in plant disease detection, supported by a bibliometric analysis of literatures from 2020 to 2025. Our analysis identifies key research hotspots, including CNN and its linked methods and technologies for image-based disease diagnosis. A dedicated section examines the groundbreaking application of LLM and LMM, including generative pre-trained transformer (GPT), bidirectional encoder representations from transformers (BERT), bootstrapping language-image pre-training (BLIP)-assisted symptom interpretation. Furthermore, this review elucidates how AI-driven GS, PS, and multi-omics selection are revolutionizing breeding programs of disease resistance traits. Finally, we critically discuss some challenges, future directions, and perspectives in plant breeding for disease resistance. The review underscores AI’s transformative role in developing disease-resistant crops to ensure food security under global change.

## 2. AI-Assisted Plant Disease Detection Based on Bibliographic Analysis

**Figure 2:**
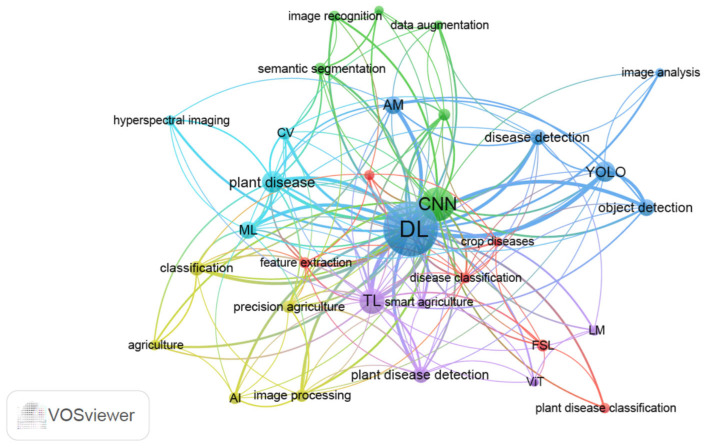
Co-occurrence network map based on authors’ keywords using VOSviewer 1.6.20 (https://www.vosviewer.com/). Nodes represent keywords with their size indicating the frequency of occurrence (larger nodes denote higher importance). Links/edges illustrate co-occurrence relationships between keywords, whereas thicker lines indicate stronger co-occurrence. Clusters are color-coded to distinguish thematic groups, with keywords of the same color belonging to the same research topic. Distance reflects the strength of association between keywords, where closer proximity implies higher relevance. Synonymous keywords “convolutional neural network”, “convolutional neural networks”, and "CNNs" are merged into the standardized form “CNN”. Different YOLO versions (e.g., YOLOv3, YOLOv5, YOLOv8) are merged into “YOLO”. AI: artificial intelligence; AM: attention mechanism; CV: computer vision; CNN: convolutional neural network; DL: deep learning; ML: machine learning; TL: transfer learning; FSL: few-shot learning; ViT: vision transformer; LM: lightweight model; YOLO: you only look once.

The application of AI in plant disease detection and diagnosis is a top priority of breeding for disease resistance. In this part, we searched published research literature on keywords including “plant disease” and “machine learning” or “plant disease” and “deep learning” from 1 January 2020 to 28 February 2025 in the PubMed database. Non-original research articles, including reviews and editorials, were excluded from the search. After deduplication, removal of irrelevant articles, and exclusion of non-science citation index (SCI) or SCI expanded indexed publications to enhance the reliability of the results, a total of 342 articles were selected to perform keyword co-occurrence analysis using VOSviewer 1.6.20 (Table S1). The 342 publications were sourced from 42 distinct journals (Table S2). Author keywords were analyzed using the full counting method, which assigns equal weight to all co-authorship or co-occurrence relationships. A threshold of five minimum co-occurrences per keyword was set to filter low-frequency terms. We also consolidated synonymous keywords by merging terms such as “convolutional neural network”, “convolutional neural networks”, and ”CNNs” into the standardized form “CNN”. The authors’ keywords were divided into six clusters from 32 keywords (Figure 2, Table S3). These keywords primarily encompassed image processing, image feature extraction, and algorithms, which are critical components in AI-driven plant disease detection systems.

Based on co-occurrence analysis metrics including link/occurrence weights and total link strength scores, DL, CNN, transfer learning (TL), and you only look once (YOLO) were top hot-spot technologies associated with plant disease detection in the recent five years (Figure 2, Table S3). Recent reviews on AI in plant breeding also underscore the significance and current research hotspots of these technologies [1,3]. CNN, a well-known DL architecture, extracts image features through convolutional layers, enables non-linear model with activation function, reduces feature dimensions with pooling layers, and conducts classification or regression tasks with fully connected layers [36]. Various DL network methods have been proposed and evolved from CNNs. Classical CNN models including LeNet [37], AlexNet [38], VGG (visual geometry group) [39], ResNet (residual network) [40], and DenseNet (densely connected convolutional networks) [41] laid the groundwork for modern DL architectures. As illustrated in Figure 2, the cutting-edge technologies including attention mechanism (AM), TL, few-shot learning (FSL), lightweight model (LM), and YOLO exhibited connections with CNN. CNNs traditionally rely on fixed-size convolutional kernels to extract local features, often overlooking global context [42]. To address the limitation, AM has emerged as a complementary or alternative approach, particularly for tasks demanding long-range dependency modeling. AM can be seamlessly integrated into CNN frameworks to enhance global feature extraction through adaptive feature refinement. A representative example is C-DenseNet, which incorporates a convolutional block attention module into the DenseNet architecture [43]. Under field conditions for wheat stripe rust disease severity grading, this hybrid model achieved a test accuracy of 97.99%, significantly surpassing classical ResNet (73.43%) and DenseNet (92.53%) baselines [43].

TL and FSL are pivotal techniques for mitigating data scarcity, enabling CNNs to achieve robust performance even with limited labeled samples. TL applies a pre-trained model learned in one task or dataset to another related task or dataset, accelerating the training process and improving performance for a new one [44]. Many CNN models such as DenseNet, SqueezeNet, ResNet, ShuffleNet, VGG, and Inception have been utilized with fine-tuning for recognizing plant leaf diseases, achieving a good accuracy of 86.93–99.7% [45,46,47,48,49]. These researchers employed fine-tuning the last layers to make the transferring task more specific to the target dataset. In recent years, early fusion and lead voting ensemble were incorporated with pre-trained CNN to reduce overfitting and improve feature extraction of plant disease images [49]. For the detection of soybean leaf diseases, Wu et al. (2023) developed an improved ConvNeXt model where AM was introduced to reduce background interference [50]. Xu et al. (2022) deployed a ViT model instead of a CNN framework to perform dual TL taking the computation load and device into consideration [51].

FSL commonly utilizes CNNs as the backbone feature extractor. As a core FSL technique, meta-learning is categorized into metric-based, model-based, and optimization-based depending on methods used for acquiring meta-knowledge [52]. For example, a semi-supervised FSL approach was induced to address leaf disease detection by incorporating domain-specific split and FSL parameters [53]. To solve the problem of limited feature extraction in FSL, Lin et al. (2022) combined cascaded multi-scale feature fusion and channel attention to detect plant diseases based on the PlantVillage dataset [54]. FREN, a feature representation enhancement network, improves feature encoding by instance embedding (Gaussian-like calibration and self-attention) and task adaptation (double pooling) [55].

LM is designed to be computationally efficient, with fewer parameters and lower memory usage, making them suitable for deployment on edge devices, including smartphones and Internet of Things sensors. Multiple LMs are based on CNNs but use techniques like depthwise separable convolutions, model pruning, and quantization to reduce complexity. For example, MobileNet, ShuffleNet, EfficientNet, lightweight transformer, and TL are developed for mobile and embedded systems, which are crucial for real-time applications like object detection and segmentation on resource-constrained devices in plant disease identification [56,57,58,59].

In object detection regarding plant diseases, YOLO is an LM known for its efficiency and real-time performance. In recent years, a series of YOLO algorithms have progressively improved objection accuracy and speed through continuous enhancements in network architecture, loss functions, data augmentation strategies, and training methods. To detect rice bacterial blight under real field conditions, the XooYOLO model was developed as an adaptation of YOLOv8 [60]. This model integrates a large selective kernel network within its backbone network, enhancing its ability to effectively identify diseases from the perspective of UAV [60]. In the domain of whole pod disease detection for common beans, Gomez et al. (2024) compared three advanced YOLO architectures combined with data augmentation techniques and found that YOLOv7 and YOLOv8 demonstrated superior performance to YOLO-NAS, with mean average precision values exceeding 95% and recall rates above 93% [61]. Furthermore, the YOLO-NAS annotation models were successfully implemented in an Android-based mobile application, demonstrating robust generalization capability with 90% classification accuracy on novel test data collected from disease-prone areas [61]. For downy mildew sporangium detection in grapevine, Yan et al. (2024) proposed AFM-YOLOv8s, an enhanced variant of YOLOv8s incorporating three novel components: a faster LM cross-stage partial module for efficient feature extraction, an adaptive cross fusion module, and a minimum point distance intersection over union loss function [62]. For tomato disease detection, Wang and Liu (2025) developed TomatoGuard-YOLO, an enhanced YOLOv10-based framework featuring: multi-path inverted residual unit for optimized multi-scale feature fusion, dynamic focusing attention for adaptive disease region detection, and focal efficient intersection over union loss for precise localization and class imbalance mitigation [63]. Therefore, YOLO architectures demonstrate high reliability and computational efficiency in plant disease detection for agricultural applications, supported by consistent performance across different field conditions and real-time deployment scenarios in multiple crop species.

## 3. Big Model in Plant Disease Detection

**Figure 3:**
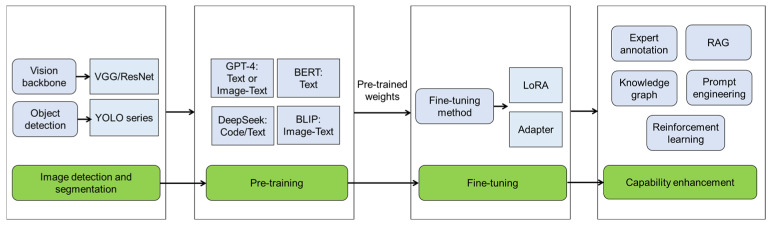
The process of image-based plant disease detection with big model. The workflow for image-based plant disease detection using big model involves image detection and segmentation through visual geometry group (VGG)/residual network (ResNet) or you only look once (YOLO) series, followed by pre-training with foundation models (large language or multi-modal models) to capture cross-model knowledge. Domain-specific adaptation is then achieved through fine-tuning technologies like low-rank adaptation (LoRA) and adapter based on pre-trained weights. To enhance diagnostic capabilities, the last phase integrates expert-annotated datasets for supervised learning, retrieval-augmented generation (RAG) for contextual reasoning, knowledge graphs for structured pathology relationships, prompt engineering for task-specific guidance, and reinforcement learning to optimize decision-making in dynamic agricultural environments. GPT: generative pre-trained transformer; BERT: bidirectional encoder representations from transformers; BLIP: bootstrapping language-image pre-training.

Although the mentioned DL models such as CNN and YOLO can provide rapid and accurate disease detection, they lack the capability to interpret the findings and provide reasonable recommendations for breeders and farmers. With the rapid advancement of computing power, big models haves further revolutionized plant disease detection through the integration of LLM or LMM with AI detection models including YOLO, VGG, and ResNet methods (Figure 3). Qing et al. (2023) made the first try and proposed a simultaneous application of YOLO and LLM for disease and pest detection [9]. The developed framework integrates a GPT-4 with YOLOPC to capture agricultural image features using edge devices and acquire real-time textual transformation of diagnosis results, and GPT-4 provides interactive suggestions for farmers, achieving 90% accuracy in reasoning for agricultural diagnostic reports with text aids [9]. The combination of YOLO and GPT-4 has also been employed to enhance pest detection in tomato cultivation [10]. Specifically, YOLOv8 was integrated for detection and segmentation tasks and GTP-4 was used to generate detailed explanations and recommendations based on detected pests [10]. Zhu et al. (2025) presented a multi-modal AI model for detecting potato early and late blight, and constructed an online potato disease detection and control platform combining the multi-modal AI model with GPT-4 [64]. CNN-transformer dual U-shaped network (CTDUNet) has been developed to merge image and text information for camellia oleifera disease and pest detection [65]. In the U-shaped branch, CTDUNet uses VGG16 as the backbone to extract comprehensive image features, whereas the LLM model BERT is employed to extract textual features and transform them into multidimensional feature spaces [65].

Unlike single-modal models, which process only text or images, multi-modal big models can integrate multiple types of information and bridge the gaps between different data modalities. ITLMLP (image-text-label multi-modal learning paradigm) is an end-to-end framework that integrates contrastive learning across image-text pairs, self-supervised image representation learning, and label semantics to optimize sample alignment within a unified multi-modal embedding space, achieving 94.84% recognition accuracy for cucumber diseases on a small dataset [8]. Nanavaty et al. (2024) presented a novel method for agricultural disease detection by synergistically combining BLIP LMM with a visual question answering framework, specifically designed to improve wheat rust identification [66]. The BLIP method enabled ResNet to interpret both complex imagery and textual inputs, therefore improving the precision and contextual relevance of its generated responses [66]. The response quality of the big model is fundamentally determined by their training data distribution, and can be systematically enhanced through pre-training [67], parameter-efficient fine-tuning technology (low-rank adaptation or adapter) [68], expert annotation [69], retrieval-augmented generation [69], structured knowledge integration (e.g., knowledge graph) [70], prompt engineering [71], and reinforcement learning from human feedback [72] (Figure 3). In particular, the potato gene-disease knowledge base/graph was constructed using LLM (spark language model 3.5), which help understand potato pathogenesis and disease resistance [70]. While knowledge graphs are well established in plant disease studies, other mentioned capability-enhancing technologies have demonstrated successful applications across diverse domains, including protein structure prediction, ontology-based plant phenotypic observations, and dietetic assessment systems, collectively evidencing their efficacy in improving the accuracy and criticality of LLM implementations.

## 4. AI-Driven Genomic Selection for Enhanced Disease Resistance

For phenotyping of plant diseases, resistance/susceptibility to diseases is usually measured in ordinal scales, count data, or percentages. While traditional linear models assume that phenotypes are continuous and normally distributed [23], these assumptions often fail to capture discrete/distorted distributions inherent in categorical or percentage-based phenotyping, non-additive genetic effects, and genotype and environment interaction. ML and DL algorithms have no restriction on the distribution of response variables and are effective in capturing non-additive and epistatic effects [20]. Representative ML and DL approaches for predicting crop disease-related traits are listed in Table 1. For wheat rust resistances, the support vector machine (SVM) regression model displayed superior or similar prediction accuracy over parametric models [20]. SVM regression can accurately predict skewed phenotypes without logarithmic or square root transformations as linear models usually address. For predicting blast, strip rust, black-streaked dwarf virus, and sheath blight resistances in rice and wheat, Liu et al. (2024) integrated kinship matrices into three ML frameworks (RF, SVM, light gradient boosting machine) to address the interference of population structure on predictions and optimize feature input through genome-wide association study-based SNP (single nucleotide polymorphism) selection to balance marker density with computational efficiency [17]. The kinship-integrated models demonstrated statistically significant superiority over both conventional ML benchmarks and deep learning architectures (deep neural network genomic prediction (DNNGP), DenseNet), achieving an average accuracy improvement of 6.8% across multiple disease resistance phenotypes [17].

According to data types of disease phenotyping values, researchers have proposed different DL methods to deal with response variables. For ordinal data, Pérez-Rodríguez et al. (2020) proposed a Bayesian regularized neural network based on a single-layer feed- forward neural network [73]. This model utilizes a data augmentation algorithm to enhance computational efficiency and demonstrates superior predictive performance compared to Bayesian linear models, particularly in predicting wheat Septoria and maize gray leaf spot diseases [73]. For count data, Montesinos-Lopez et al. (2021) introduced a Poisson deep neural network (PDNN) method, which leveraged multiple nonlinear transformations in hidden layers to capture complex data patterns [74]. This approach demonstrated high prediction accuracy for wheat Fusarium head blight severity. To address multivariate count data, Montesinos-López et al. (2020) developed a multivariate PDNN model for the simultaneous genomic prediction of multiple count outcomes [22]. DL framework achieved strong prediction performance for disease resistance traits, attributed to its ability to capture nonlinear patterns. This is accomplished by incorporating multiple hidden layers in the network architecture, which apply nonlinear transformations to the data, enabling the model to train intricate patterns effectively.

In recent years, transformers have been applied to genomic prediction for plant diseases. GPTransformer, a transformer-based genomic prediction model, can identify the inter-relation among markers, especially with Hardy-Weinberg encoding, and performed with good accuracy for barley Fusarium head blight severity using genotypic and phenotypic data [18]. Chen et al. (2024) found that the attention network surpassed RF, multilayer perceptron, and modified CNN in sugarcane disease prediction tasks, showing higher precision and lower mean square error [21]. Its performance was comparable to parametric statistical models in most cases. Furthermore, integrating Bayesian prior knowledge into the attention network resulted in the most stable prediction accuracy across cross-validation folds, exhibiting minimal variability compared to other methods [21].

## 5. Leveraging AI for Phenomic Selection of Disease Resistance Phenotypes

The convergence Internet-of-Things-enabled plant-of-things technologies and AI has propelled plant phenomics into a new era, enabling precision breeding through high-throughput measurement of complex traits. This paradigm shift is exemplified by the emergence of PS as an alternative or complement to GS for grain yield and yield-related traits in wheat, triticale, potato, and rapeseed [27,28,76,77,78,79].

PS allows rapid gathering of spectral data across all growth stages in the field with the aid of portable spectrometers, UAV equipped with hyperspectral or multispectral cameras, and satellite remote sensing, which enables nondestructive measurements of traits especially for resistance to disease. For disease-resistant traits, linear regression models with marker matrices achieved high predictive abilities, while those of phenomic prediction using NIRS predictors were very low [77]. The limited ability of linear models to capture temporal and spatial features related to disease dynamics may explain the low accuracy of PS. ML and DL techniques are primarily employed for automated feature extraction and analysis of HTP-base traits. DeSalvio et al. (2022) integrated five ML methods with 36 vegetation indices from UAV-acquired mosaic images to detect maize southern rust, achieving higher prediction accuracies and lower root mean squared error compared to a general linear model [28] (Table 1). This study demonstrated the potential of a simple RGB camera on a UAV to capture spatio-temporal data, which can be used to train ML models for predicting important phenomena such as southern rust progression. Thapa et al. (2024) observed ground-based hyperspectral imaging in conjugation with DL based one-dimensional CNN model as a novel avenue to estimate wheat Fusarium head blight-related traits with prediction accuracy comparable to traditional GS models, suggesting a great potential for phenomic prediction in developing Fusarium head blight-resistant cultivars [19]. Except for prediction models, several factors such as genetic architecture, HTP-based features, environments, sizes and compositions of the training set also impacted the accuracy of PS [76,77,80].

Compared to GS, PS is still in its infancy. In GS, genomic markers are static and independent of environmental influences. In contrast, phenomic markers are dynamic and environment-specific, enabling them to capture variations across different environments and contribute to a deeper understanding of phenotypic plasticity. While PS demonstrates clear strengths, it still faces challenges such as sensor costs, data processing complexities (including feature extraction and integration of multidimensional imaging and spectral data), and dynamic modeling of temporal trait variations. Addressing these limitations requires simultaneous advancements in hardware innovation and AI algorithm optimization to enhance scalability, data interpretability, and real-time adaptability.

## 6. Leveraging AI to Align Multi-Omics Signatures with Disease Resistance Phenotypes

**Figure 4:**
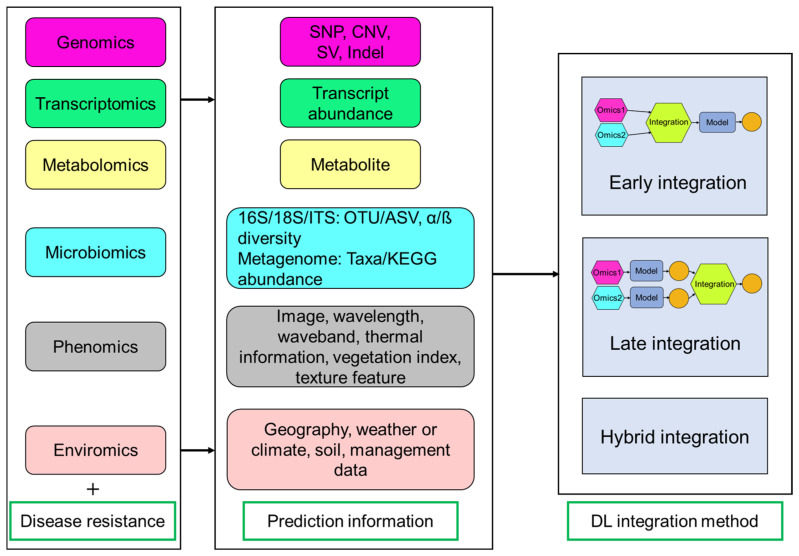
Overview of multi-omics data and integration methods for predicting disease-resistant phenotypes. Multi-omics prediction includes different omics data (e.g., genomics, transcriptomics, metabolomics, microbiomics, phenomics, and enviromics) and disease-resistant phenotypic data. Deep learning (DL) integration methods are divided into three categories (early, late, and hybrid integration). Early integration is an end-to-end method that combination is conducted before model prediction. Late integration happens after model prediction and involves integrating individual prediction results. Hybrid approaches use both early and late integration methods. SNP: single nucleotide polymorphism; CNV: copy number variation; SV: structural variation; Indel: insertion-deletion; ITS: internal transcribed spacer; OTU: operational taxonomic unit; ASV: amplicon sequence variant.

Disease-resistant phenotype is a complex polygenic output involving interactions of multiple layers including genomic markers, gene expression, metabolites, microorganisms, and more. The key predictive features identified through multi-omics analysis are summarized in Figure 4. The utilization of mono-omics approach does not provide sufficient knowledge to understand the plant-pathogen interactions. The integration of transcriptomic profiles, metabolic signatures, and hyperspectral images has reported a high accuracy in detecting four prevalent diseases based on an explainable gradient-based approach EG-CNN model [81]. Therefore, multi-omics integration holds promise for systematically enhancing plant disease resistance by uncovering endophenotypes.

DL is uniquely equipped to address the dual challenges of high dimensionality and multimodality inherent in multi-omics datasets, leveraging its capacity to process massive-scale data, resolve structural complexities, and extract latent patterns across heterogeneous modalities. Key multi-omics integration methods include early integration (combining data at the start), late integration (merging results at the end), and hybrid methods (mixing both approaches) [82,83] (Figure 4). DNNGP is a DL-based GS technique including three CNN layers (one batch normalization layer and two dropout layers) that can directly integrate diverse multi-omics data during the initial input phase to predict universal plant phenotypes [4]. This approach dynamically captures features from heterogeneous data sources through integrated learning and is classified as an early integration model. Dual-extraction modeling (DEM) is a multi-modal and mixed integration method that can capture representative features from different heterogeneous omics data, enabling phenotypic prediction, such as yield-related traits in maize and disease resistance in rice [75]. DEM operates through two sequential phases: initially processing single-omics data and labels through a multi-head self-attention network for feature extraction, followed by integrating the resulting latent features into a joint matrix [75]. This matrix undergoes noise filtering and dimensionality reduction via a subsequent self-attention network to optimize cross-omics analysis. CustOmics (customizable architecture for multi-omics integration) is a mixed integration model that combines the advantages of early and late integration through deep neural networks to model cross-modal interactions, which has been used in classification tasks for straight head susceptibility and blast resistance from rice [75,83].

## 7. Challenges and Perspectives

Although significant progress has been made in the application of AI in plant disease resistance breeding, there are still some challenges in terms of data, model, and privacy to overcome. A major challenge is the lack of high-quality, large-scale, and standardized datasets, which significantly impacted the training of AI models. The balance between data sharing and privacy is challenging. Model interpretability, visualization and transparency of model outcomes, computational power, and low acceptance of new technologies also hinder the application and development of AI in agriculture.

These challenges also bring new opportunities and innovation directions for scientists and practitioners. Future directions of disease resistance breeding should primarily focus on developing more efficient algorithms and multimodal AI models to improve the prediction accuracy of disease phenotypes. Recent advances in DL, such as transformer-based architectures like GPTransformer and cross-modal attention modules, have shown promise in modeling complex genotype-phenotype relationships in plant diseases [18,84]. Additionally, integrating multi-omics data (genomics, transcriptomics, microbiomics, and phenomics) through AI-driven approaches can enhance predictive power and biological interpretability [4,75,83]. Developing explainable AI can improve model transparency and finally build trust among end-users. Techniques like Shapley additive explanations (SHAP), local interpretable model-agnostic explanations, and integrated gradient can elucidate the decision-making process of AI models. These methods have been successfully applied in agricultural AI systems to provide actionable insights. For instance, SHAP method can effectively and accurately extract critical wavelength features from hyperspectral imaging data, enabling dilated CNN to achieve reasonable performance in predicting soybean rust [85]. The development of smart breeding platforms can help overcome the low adoption rate of new technologies for breeders and farmers. AI platforms such as Smart Breeding Platform and AutoGP, and project initiatives like CropGPT are paving the way for data-driven and scalable solutions for plant breeding [86,87,88]. The main limitation of these platforms is their lack of LLM integration and insufficient data coverage, depriving users of critical features such as interactive question-answering, intelligent search, and historical data insights. 

Federated learning (FL) is a privacy-preserving distributed ML approach that enables decentralized devices or data centers (e.g., smartphones, edge servers, farm systems) to collaboratively train a shared global model while maintaining raw data locally, thereby addressing both data privacy and insufficiency challenges through secure and shared learning. For human disease diagnosis, FL models trained collaboratively on multi-omics data can achieve high classification accuracy and maintain robust performance despite heterogeneity across client sites [89,90]. Although FL applications in plant disease detection remain less explored compared to human disease studies, preliminary research has been reported. Kabala et al. (2023) evaluated five CNN models and two ViT models within an FL framework using the PlantVillage image dataset and identified ResNet50 as the highest-performing model with an accuracy approaching 99.5% [91]. This study demonstrates that effective FL implementation requires a careful selection of AI methods to balance model performance with computational efficiency, along with the optimal configuration of key parameters including client population size, communication round frequency, local iteration counts, and dataset quality metrics. In addition, developing LMs with reduced parameter counts and lower computational demands is essential for FL scenarios characterized by data heterogeneity and communication constraints.

**Figure 5:**
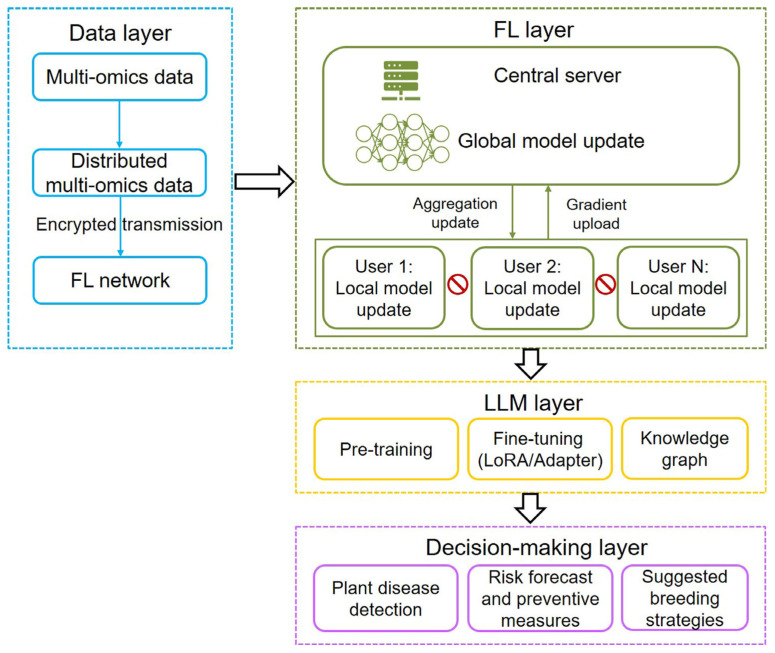
Architecture of the proposed framework combining multi-omics data, federated learning (FL), and large language model (LLM) technologies. The framework contains multi-omics data layer, FL layer, LLM layer, and decision-making layer. In FL, models are trained across decentralized devices while keeping data localized, with a central server aggregating only the model parameter updates. Local models can be regression prediction model or image detection models such as ResNet (residual network) or YOLO (you only look once), which allow resistant-phenotype prediction and plant disease detection using multi-omics-based structured and unstructured data, respectively. LLM training involves three key components: pre-training, fine-tuning, and integration with knowledge graphs. LoRA: low-rank adaptation.

The scarcity of high-quality data is also a big challenge for training LLM. To solve the problem, current scholarly efforts have focused on the combination of FL with LLM. In general, three integration frameworks include using LLM techniques in FL, applying FL methods to LLM, and merging LLM and FL as a unified system (federated LLM) [67]. Existing FL-LLM architectures predominantly focus on the collaborative training of a globally shared model, largely neglecting the critical aspect of model personalization, such as plant disease detection and prediction. In the future, innovative breeding technologies, including multi-omics data, FL, AI algorithms, and LLM, will be integrated into a smart breeding pipeline for plant disease detection and prediction (Figure 5). The pipeline will balance both data privacy and plant disease management and breeding strategy suggestion. In general, this pipeline will comprise four hierarchical layers: multi-omics data integration, FL, LLM processing, and decision-making. It adopts a distributed architecture design, enabling nodes such as different users to process multi-modal data locally while achieving federated aggregation through encrypted gradient uploads. The training pipeline of LLMs is typically structured around three core phases: pre-training on large-scale corpora to acquire general linguistic patterns, task-specific fine-tuning to adapt to downstream applications, and knowledge graph grounding to enhance factual accuracy and reasoning capabilities. Decision-making components comprise plant disease detection, risk forecast and preventive measures, and suggested breeding strategies. With the rise of AI agents, as exemplified by their application in fully automated multi-omics analyses [92], agricultural decision-making is poised for transformative innovation. At the decision-making layer, AI agents will be integrated to enhance analytical capabilities and optimize strategic planning. By incorporating predictive modeling, AI agents will assist policymakers and farm managers in making data-driven decisions, from resource allocation to plant disease intervention protocols, ensuring more efficient and sustainable agricultural practices. This pipeline effectively safeguards data sovereignty while facilitating knowledge sharing, thereby assisting breeders and farmers in making intelligent decisions on disease management and breeding strategies for disease-resistant varieties. However, constructing such an integrated collaborative platform presents significant challenges. Effective collaboration across institutions and enterprises requires scalable information management systems for large-team coordination, robust mechanisms for knowledge protection and sharing, cultivating interdisciplinary experts, training skilled farmers (operators) for precise results, and long-term stable funding. 

## Figures and Tables

**Table 1:** Representative machine learning and deep learning approaches for predicting disease-related traits in crops using genomics, phenomics, and multi-omics data.

Omics Type	Crop Species	Disease ^1^	Model ^2^	Type	Accuracy ^3^	Reference
Genomics	Rice, wheat	RB, RBSDV, RSB, WB, WSR	RF, SVM, lightGBM, RFC_K, SVC_K, lightGBM_K, DNNGP, DenseNet	Classification	0.71–0.98	[17]
	Barley	FHB	GPTransformer, RFCNN, DT	Regression	0.34–0.62	[18]
	Wheat	SR	SVM, SVMR	Classification, regression	-	[20]
	Sugarcane	Smut, PRR	Attention network, RF, MLP, modified CNN	Regression	0.28–0.49	[21]
	Wheat	SN, PTR, SB	MPDN, UPDN, GPR	Regression	0.33–0.66	[22]
	Wheat, Maize	*Septoria*, GLS	BRNNO	Regression	0.31–0.87	[73]
	Wheat	FHB	PDNN, DNN, GPER	Regression	0.35–0.81	[74]
Phenomics	Wheat	FHB-related traits	One-dimensional CNN	Regression	0.45–0.55	[19]
	Maize	Southern rust	RF, SVM (radial and linear kernel), EN, KNN	Regression	-	[28]
Multi-Omics	Rice	SS, BR	DEM	Classification	0.62–0.70	[75]
	Rice	SS, BR	CustOmics	Classification	0.50–0.60	[75]

^1^ FHB: *Fusarium* head blight; SR: stripe rust; PRR: Pachymetra root rot; SN: *Parastagonospora nodorum*; PTR: *Pyrenophora* tritici-repentis; SB: *Bipolaris sorokiniana*; RB: rice blast, RBSDV: rice black-streaked dwarf virus; RSB: rice sheath blight; WB: wheat blast; WSR: wheat stripe rust; GLS: gray leaf spot; SS: straight head susceptibility; BR: blast resistance. ^2^ RFCNN: residual fully connected neural network; DT: decision tree; SVM: support vector machine, SVMR: support vector machine regression; RF: random forest; MLP: multilayer perceptron; modified CNN: modified convolutional neural network; MPDN: multivariate Poisson deep neural network; UPDN: univariate Poisson deep neural network; GPR: univariate generalized Poisson elastic net regression; lightGBM: light gradient boosting machine; RFC_K: random forest classification plus kinship; SVC_K: support vector classification plus kinship; lightGBM_K: light gradient boosting machine plus kinship; DNNGP: deep neural network genomic prediction. DenseNet: densely connected convolutional networks; BRNNO: Bayesian regularized neural network; PDNN: Poisson deep neural network; DNN: normal deep neural network; GPER: generalized Poisson elastic net regression; CNN: convolutional neural network; EN: elastic net; KNN: K-nearest neighbors; DEM: dual-extraction modeling; CustOmics: customizable architecture for multi-omics integration. ^3^ The accuracy denotes Pearson correlation coefficient [18,21,22], coefficient of determination [19], Spearman correlation coefficient [73,74], and mean accuracy [17,75].

## Data Availability

No new data were created in this study. Data sharing is not applicable to this article.

## Supplementary Materials

The following supporting information can be downloaded at: https://www.mdpi.com/article/10.3390/ijms26115324/s1.

## References

[1] Zhu H., Lin C., Liu G., Wang D., Qin S., Li A., Xu J.L., He Y. (2024). Intelligent agriculture: Deep learning in UAV-based remote sensing imagery for crop diseases and pests detection. Front. Plant Sci..

[2] Wallace J.G., Rodgers-Melnick E., Buckler E.S. (2018). On the road to Breeding 4.0: Unraveling the good, the bad, and the boring of crop quantitative genomics. Annu. Rev. Genet..

[3] Farooq M.A., Gao S., Hassan M.A., Huang Z., Rasheed A., Hearne S., Prasanna B., Li X., Li H. (2024). Artificial intelligence in plant breeding. Trends Genet..

[4] Wang K., Abid M.A., Rasheed A., Crossa J., Hearne S., Li H. (2023). DNNGP, a deep neural network-based method for genomic prediction using multi-omics data in plants. Mol. Plant.

[5] Vaswani A., Shazeer N., Parmar N., Uszkoreit J., Jones L., Gomez A.N., Kaiser Ł., Polosukhin I. (2017). Attention is all you need. Adv. Neural Inf. Process. Syst..

[6] Wang Y., Zhang Y., Liu Y., Yang Z., Xiong H., Wang Y., Lin J., Yang Y. (2023). From instructions to intrinsic human values: A survey of alignment goals for big models. arXiv.

[7] Bommasani R., Hudson D.A., Adeli E., Altman R., Arora S., von Arx S., Bernstein M.S., Bohg J., Bosselut A., Brunskill E. (2022). On the opportunities and risks of foundation models. arXiv.

[8] Cao Y.Y., Chen L., Yuan Y., Sun G.L. (2023). Cucumber disease recognition with small samples using image-text-label based multi-modal language model. Comput. Electron. Agric..

[9] Qing J., Deng X., Lan Y., Li Z. (2023). GPT-aided diagnosis on agricultural image based on a new light YOLOPC. Comput. Electron. Agric..

[10] Sahin Y.S., Gençer N.S., Şahin H. (2025). Integrating AI detection and language models for real-time pest management in tomato cultivation. Front. Plant Sci..

[11] Holzinger A., Saranti A., Angerschmid A., Retzlaff C.O., Gronauer A., Pejakovic V., Medel-Jimenez F., Krexner T., Gollob C., Stampfer K. (2022). Digital transformation in smart farm and forest operations needs human-centered AI: Challenges and future directions. Sensors.

[12] Zhang W., Boyle K., Brule-Babel A., Fedak G., Gao P., Djama Z.R., Polley B., Cuthbert R., Randhawa H., Graf R. (2021). Evaluation of genomic prediction for Fusarium head blight resistance with a multi-parental population. Biology.

[13] Semagn K., Iqbal M., Jarquin D., Crossa J., Howard R., Ciechanowska I., Henriquez M.A., Randhawa H., Aboukhaddour R., McCallum B.D. (2022). Genomic predictions for common bunt, FHB, stripe rust, leaf rust, and leaf spotting resistance in spring wheat. Genes.

[14] Butoto E.N., Brewer J.C., Holland J.B. (2022). Empirical comparison of genomic and phenotypic selection for resistance to Fusarium ear rot and fumonisin contamination in maize. Theor. Appl. Genet..

[15] Islam M.S., McCord P.H., Olatoye M.O., Qin L., Sood S., Lipka A.E., Todd J.R. (2021). Experimental evaluation of genomic selection prediction for rust resistance in sugarcane. Plant Genome.

[16] Pincot D.D.A., Hardigan M.A., Cole G.S., Famula R.A., Henry P.M., Gordon T.R., Knapp S.J. (2020). Accuracy of genomic selection and long-term genetic gain for resistance to Verticillium wilt in strawberry. Plant Genome.

[17] Liu Q., Zuo S.-M., Peng S., Zhang H., Peng Y., Li W., Xiong Y., Lin R., Feng Z., Li H. (2024). Development of machine learning methods for accurate prediction of plant disease resistance. Engineering.

[18] Jubair S., Tucker J.R., Henderson N., Hiebert C.W., Badea A., Domaratzki M., Fernando W.G.D. (2021). GPTransformer: A transformer-based deep learning method for predicting Fusarium related traits in barley. Front. Plant Sci..

[19] Thapa S., Gill H.S., Halder J., Rana A., Ali S., Maimaitijiang M., Gill U., Bernardo A., St Amand P., Bai G. (2024). Integrating genomics, phenomics, and deep learning improves the predictive ability for Fusarium head blight-related traits in winter wheat. Plant Genome.

[20] Merrick L.F., Lozada D.N., Chen X., Carter A.H. (2022). Classification and regression models for genomic selection of skewed phenotypes: A case for disease resistance in winter wheat (*Triticum aestivum* L.). Front. Genet..

[21] Chen C., Bhuiyan S.A., Ross E., Powell O., Dinglasan E., Wei X., Atkin F., Deomano E., Hayes B. (2024). Genomic prediction for sugarcane diseases including hybrid Bayesian-machine learning approaches. Front. Plant Sci..

[22] Montesinos-López O.A., Montesinos-López J.C., Singh P., Lozano-Ramirez N., Barrón-López A., Montesinos-López A., Crossa J. (2020). A multivariate Poisson deep learning model for genomic prediction of count data. G3 Genes|Genomes|Genet..

[23] González-Camacho J.M., Ornella L., Pérez-Rodríguez P., Gianola D., Dreisigacker S., Crossa J. (2018). Applications of machine learning methods to genomic selection in breeding wheat for rust resistance. Plant Genome.

[24] Rincent R., Charpentier J.P., Faivre-Rampant P., Paux E., Le Gouis J., Bastien C., Segura V. (2018). Phenomic selection is a low-cost and high-throughput method based on indirect predictions: Proof of concept on wheat and poplar. G3 Genes|Genomes|Genet..

[25] Galán R.J., Bernal-Vasquez A.M., Jebsen C., Piepho H.P., Thorwarth P., Steffan P., Gordillo A., Miedaner T. (2020). Integration of genotypic, hyperspectral, and phenotypic data to improve biomass yield prediction in hybrid rye. Theor. Appl. Genet..

[26] Maggiorelli A., Baig N., Prigge V., Bruckmüller J., Stich B. (2024). Using drone-retrieved multispectral data for phenomic selection in potato breeding. Theor. Appl. Genet..

[27] Jackson R., Buntjer J.B., Bentley A.R., Lage J., Byrne E., Burt C., Jack P., Berry S., Flatman E., Poupard B. (2023). Phenomic and genomic prediction of yield on multiple locations in winter wheat. Front. Genet..

[28] DeSalvio A.J., Adak A., Murray S.C., Wilde S.C., Isakeit T. (2022). Phenomic data-facilitated rust and senescence prediction in maize using machine learning algorithms. Sci. Rep..

[29] Adak A., Kang M., Anderson S.L., Murray S.C., Jarquin D., Wong R.K.W., Katzfuß M. (2023). Phenomic data-driven biological prediction of maize through field-based high-throughput phenotyping integration with genomic data. J. Exp. Bot..

[30] Adak A., DeSalvio A.J., Arik M.A., Murray S.C. (2024). Field-based high-throughput phenotyping enhances phenomic and genomic predictions for grain yield and plant height across years in maize. G3 Genes|Genomes|Genet..

[31] Togninalli M., Wang X., Kucera T., Shrestha S., Juliana P., Mondal S., Pinto F., Govindan V., Crespo-Herrera L., Huerta-Espino J. (2023). Multi-modal deep learning improves grain yield prediction in wheat breeding by fusing genomics and phenomics. Bioinformatics.

[32] Maimaitijiang M., Sagan V., Sidike P., Hartling S., Esposito F., Fritschi F.B. (2020). Soybean yield prediction from UAV using multimodal data fusion and deep learning. Remote Sens. Environ..

[33] Kaushal S., Gill H.S., Billah M.M., Khan S.N., Halder J., Bernardo A., Amand P.S., Bai G., Glover K., Maimaitijiang M. (2024). Enhancing the potential of phenomic and genomic prediction in winter wheat breeding using high-throughput phenotyping and deep learning. Front. Plant Sci..

[34] Lecun Y., Bengio Y., Hinton G. (2015). Deep learning. Nature.

[35] Kang M., Ko E., Mersha T.B. (2022). A roadmap for multi-omics data integration using deep learning. Brief. Bioinform..

[36] Gu J., Wang Z., Kuen J., Ma L., Shahroudy A., Shuai B., Liu T., Wang X., Wang G., Cai J. (2018). Recent advances in convolutional neural networks. Pattern Recognit..

[37] LeCun Y., Bottou L., Bengio Y., Haffner P. (1998). Gradient-based learning applied to document recognition. Proc. IEEE.

[38] Krizhevsky A., Sutskever I., Hinton G.E. (2017). Imagenet classification with deep convolutional neural networks. Commun. ACM.

[39] Simonyan K., Zisserman A. (2014). Very deep convolutional networks for large-scale image recognition. arXiv.

[40] He K., Zhang X., Ren S., Sun J. Deep residual learning for image recognition. Proceedings of the IEEE Conference on Computer Vision and Pattern Recognition.

[41] Huang G., Liu Z., Van Der Maaten L., Weinberger K.Q. Densely connected convolutional networks. Proceedings of the IEEE Conference on Computer Vision and Pattern Recognition.

[42] Choi S.R., Lee M. (2023). Transformer architecture and attention mechanisms in genome data analysis: A comprehensive review. Biology.

[43] Mi Z., Zhang X., Su J., Han D., Su B. (2020). Wheat stripe rust grading by deep learning with attention mechanism and images from mobile devices. Front. Plant Sci..

[44] Pan S.J., Yang Q. (2010). A survey on transfer learning. IEEE Trans. Knowl. Data Eng..

[45] Gu Y.H., Yin H., Jin D., Park J.H., Yoo S.J. (2021). Image-based hot pepper disease and pest diagnosis using transfer learning and fine-tuning. Front. Plant Sci..

[46] Krishnamoorthy N., Prasad L.N., Kumar C.P., Subedi B., Abraha H.B., Easwaramoorthy S.V. (2021). Rice leaf diseases prediction using deep neural networks with transfer learning. Environ. Res..

[47] Shahoveisi F., Taheri Gorji H., Shahabi S., Hosseinirad S., Markell S., Vasefi F. (2023). Application of image processing and transfer learning for the detection of rust disease. Sci. Rep..

[48] Kini A.S., Prema K.V., Pai S.N. (2024). Early stage black pepper leaf disease prediction based on transfer learning using ConvNets. Sci. Rep..

[49] Shafik W., Tufail A., De Silva Liyanage C., Apong R.A.A.H.M. (2024). Using transfer learning-based plant disease classification and detection for sustainable agriculture. BMC Plant Biol..

[50] Wu Q., Ma X., Liu H., Bi C., Yu H., Liang M., Zhang J., Li Q., Tang Y., Ye G. (2023). A classification method for soybean leaf diseases based on an improved ConvNeXt model. Sci. Rep..

[51] Xu M., Yoon S., Jeong Y., Park D.S. (2022). Transfer learning for versatile plant disease recognition with limited data. Front. Plant Sci..

[52] Wu X., Deng H., Wang Q., Lei L., Gao Y., Hao G. (2023). Meta-learning shows great potential in plant disease recognition under few available samples. Plant J..

[53] Li Y., Chao X. (2021). Semi-supervised few-shot learning approach for plant diseases recognition. Plant Methods.

[54] Lin H., Tse R., Tang S.K., Qiang Z.P., Pau G. (2022). Few-shot learning approach with multi-scale feature fusion and attention for plant disease recognition. Front. Plant Sci..

[55] Lin H., Qiang Z., Tse R., Tang S.K., Pau G. (2024). A few-shot learning method for tobacco abnormality identification. Front. Plant Sci..

[56] Li G., Wang Y., Zhao Q., Yuan P., Chang B. (2023). PMVT: A lightweight vision transformer for plant disease identification on mobile devices. Front. Plant Sci..

[57] Zhang E., Zhang N., Li F., Lv C. (2024). A lightweight dual-attention network for tomato leaf disease identification. Front. Plant Sci..

[58] Prince R.H., Mamun A.A., Peyal H.I., Miraz S., Nahiduzzaman M., Khandakar A., Ayari M.A. (2024). CSXAI: A lightweight 2D CNN-SVM model for detection and classification of various crop diseases with explainable AI visualization. Front. Plant Sci..

[59] Mazumder M.K.A., Mridha M.F., Alfarhood S., Safran M., Abdullah-Al-Jubair M., Che D. (2024). A robust and light-weight transfer learning-based architecture for accurate detection of leaf diseases across multiple plants using less amount of images. Front. Plant Sci..

[60] Pan P., Guo W., Zheng X., Hu L., Zhou G., Zhang J. (2023). Xoo-YOLO: A detection method for wild rice bacterial blight in the field from the perspective of unmanned aerial vehicles. Front. Plant Sci..

[61] Gómez D., Selvaraj M.G., Casas J., Mathiyazhagan K., Rodriguez M., Assefa T., Mlaki A., Nyakunga G., Kato F., Mukankusi C. (2024). Advancing common bean (*Phaseolus vulgaris* L.) disease detection with YOLO driven deep learning to enhance agricultural AI. Sci. Rep..

[62] Yan C., Liang Z., Yin L., Wei S., Tian Q., Li Y., Cheng H., Liu J., Yu Q., Zhao G. (2024). AFM-YOLOv8s: An accurate, fast, and highly robust model for detection of sporangia of Plasmopara viticola with various morphological variants. Plant Phenomics.

[63] Wang X., Liu J. (2025). TomatoGuard-YOLO: A novel efficient tomato disease detection method. Front. Plant Sci..

[64] Zhu H., Shi W., Guo X., Lyu S., Yang R., Han Z. (2025). Potato disease detection and prevention using multimodal AI and large language model. Comput. Electron. Agric..

[65] Guo R., Zhang R., Zhou H., Xie T., Peng Y., Chen X., Yu G., Wan F., Li L., Zhang Y. (2024). CTDUNet: A multimodal CNN-transformer dual U-shaped network with coordinate space attention for camellia oleifera pests and diseases segmentation in complex environments. Plants.

[66] Nanavaty A., Sharma R., Pandita B., Goyal O., Rallapalli S., Mandal M., Singh V.K., Narang P., Chamola V. (2024). Integrating deep learning for visual question answering in agricultural disease diagnostics: Case study of wheat rust. Sci. Rep..

[67] Chen C., Feng X., Li Y., Lyu L., Zhou J., Zheng X., Yin J. (2024). Integration of large language models and federated learning. Patterns.

[68] Zeng S., Wang D., Jiang L., Xu D. (2024). Parameter-efficient fine-tuning on large protein language models improves signal peptide prediction. Genome Res..

[69] Kainer D. (2025). The effectiveness of large language models with RAG for auto-annotating trait and phenotype descriptions. Biol. Methods Protoc..

[70] Xie C., Gao J., Chen J., Zhao X. (2024). PotatoG-DKB: A potato gene-disease knowledge base mined from biological literature. PeerJ.

[71] Azimi I., Qi M., Wang L., Rahmani A.M., Li Y. (2025). Evaluation of LLMs accuracy and consistency in the registered dietitian exam through prompt engineering and knowledge retrieval. Sci. Rep..

[72] Ouyang L., Wu J., Jiang X., Almeida D., Wainwright C.L., Mishkin P., Zhang C., Agarwal S., Slama K., Ray A. (2022). Training language models to follow instructions with human feedback. arXiv.

[73] Pérez-Rodríguez P., Flores-Galarza S., Vaquera-Huerta H., Del Valle-Paniagua D.H., Montesinos-López O.A., Crossa J. (2020). Genome-based prediction of Bayesian linear and non-linear regression models for ordinal data. Plant Genome.

[74] Montesinos-López O.A., Montesinos-López J.C., Salazar E., Barron J.A., Montesinos-López A., Buenrostro-Mariscal R., Crossa J. (2021). Application of a Poisson deep neural network model for the prediction of count data in genome-based prediction. Plant Genome.

[75] Ren Y., Wu C., Zhou H., Hu X., Miao Z. (2024). Dual-extraction modeling: A multi-modal deep-learning architecture for phenotypic prediction and functional gene mining of complex traits. Plant Commun..

[76] Dallinger H.G., Löschenberger F., Bistrich H., Ametz C., Hetzendorfer H., Morales L., Michel S., Buerstmayr H. (2023). Predictor bias in genomic and phenomic selection. Theor. Appl. Genet..

[77] Zhu X., Maurer H.P., Jenz M., Hahn V., Ruckelshausen A., Leiser W.L., Würschum T. (2022). The performance of phenomic selection depends on the genetic architecture of the target trait. Theor. Appl. Genet..

[78] Roscher-Ehrig L., Weber S.E., Abbadi A., Malenica M., Abel S., Hemker R., Snowdon R.J., Wittkop B., Stahl A. (2024). Phenomic selection for hybrid rapeseed breeding. Plant Phenomics.

[79] DeSalvio A.J., Adak A., Murray S.C., Jarquín D., Winans N.D., Crozier D., Rooney W.L. (2024). Near-infrared reflectance spectroscopy phenomic prediction can perform similarly to genomic prediction of maize agronomic traits across environments. Plant Genome.

[80] Robert P., Goudemand E., Auzanneau J., Oury F.X., Rolland B., Heumez E., Bouchet S., Caillebotte A., Mary-Huard T., Le Gouis J. (2022). Phenomic selection in wheat breeding: Prediction of the genotype-by-environment interaction in multi-environment breeding trials. Theor. Appl. Genet..

[81] Shoaib M., Shah B., Sayed N., Ali F., Ullah R., Hussain I. (2023). Deep learning for plant bioinformatics: An explainable gradient-based approach for disease detection. Front. Plant Sci..

[82] Tang X., Prodduturi N., Thompson K.J., Weinshilboum R., O’Sullivan C.C., Boughey J.C., Tizhoosh H.R., Klee E.W., Wang L., Goetz M.P. (2024). OmicsFootPrint: A framework to integrate and interpret multi-omics data using circular images and deep neural networks. Nucleic Acids Res..

[83] Benkirane H., Pradat Y., Michiels S., Cournède P.H. (2023). CustOmics: A versatile deep-learning based strategy for multi-omics integration. PLoS Comput. Biol..

[84] Liu R., Guo X., Zhu H., Wang L. (2025). A text-speech multimodal Chinese named entity recognition model for crop diseases and pests. Sci. Rep..

[85] Feng J., Zhang S., Zhai Z., Yu H., Xu H. (2024). DC2Net: An Asian soybean rust detection model based on hyperspectral imaging and deep learning. Plant Phenomics.

[86] Li H., Li X., Zhang P., Feng Y., Mi J., Gao S., Sheng L., Ali M., Yang Z., Li L. (2024). Smart Breeding Platform: A web-based tool for high-throughput population genetics, phenomics, and genomic selection. Mol. Plant.

[87] Wu H., Han R., Zhao L., Liu M., Chen H., Li W., Li L. (2025). AutoGP: An intelligent breeding platform for enhancing maize genomic selection. Plant Commun..

[88] Zhu W., Han R., Shang X., Zhou T., Liang C., Qin X., Chen H., Feng Z., Zhang H., Fan X. (2024). The CropGPT project: Call for a global, coordinated effort in precision design breeding driven by AI using biological big data. Mol. Plant.

[89] Danek B.P., Makarious M.B., Dadu A., Vitale D., Lee P.S., Singleton A.B., Nalls M.A., Sun J., Faghri F. (2024). Federated learning for multi-omics: A performance evaluation in *Parkinson*’s disease. Patterns.

[90] Wang Q., He M., Guo L., Chai H. (2023). AFEI: Adaptive optimized vertical federated learning for heterogeneous multi-omics data integration. Brief. Bioinform..

[91] Kabala D.M., Hafiane A., Bobelin L., Canals R. (2023). Image-based crop disease detection with federated learning. Sci. Rep..

[92] Zhou J., Zhang B., Li G., Chen X., Li H., Xu X., Chen S., He W., Xu C., Liu L. (2024). An AI agent for fully automated multi-omic analyses. Adv. Sci..

